# A simple fruit and vegetable score is a valid tool to assess actual fruit and vegetable intake

**DOI:** 10.1017/S0007114523001125

**Published:** 2023-12-14

**Authors:** Giulia Pastori, Inge D. Brouwer, Edith J. M. Feskens, Le Thi Huong, Folake O. Samuel, Le Thi Thanh Xuan, Oluyemisi F. Shittu, Toluwalope E. Eyinla, Elise F. Talsma

**Affiliations:** 1 Department of Global Nutrition, Division of Human Nutrition and Health, Wageningen University and Research, 6700 AA Wageningen, the Netherlands; 2 International Food Policy Research Institute, Washington, DC, USA; 3 Institute for Preventive Medicine and Public Health, Hanoi Medical University, Hanoi, Vietnam; 4 Department of Human Nutrition and Dietetics, University of Ibadan, Ibadan, Nigeria

**Keywords:** Fruit and vegetable component of the Global Dietary Recommendation score, Indicator, Diet quality, Dietary assessment, Validation, Consumption, Food groups

## Abstract

With the recent growing interest in improving fruit and vegetable intake for better health and limited research resources in many settings, simple-to-administer and low-priced indicators are essential tools for monitoring fruit and vegetable intake at the population level. A potential candidate indicator is the fruit and vegetable component of the Global Dietary Recommendation score (FV-GDR) based on data collected using the Diet Quality Questionnaire (DQQ). We investigated the relative validity of FV-GDR collected with the DQQ to measure fruit and vegetable intake by comparison with a 24-h recall (24hR) as a reference collected from 620 Vietnamese and 630 Nigerian adults in 2021. We found proportional differences in the prevalence of intake of vitamin A-rich vegetables, other vegetables and other fruits in Vietnam and all vegetable food groups in Nigeria. In both countries, we found a small difference in the total FV-GDR from DQQ compared with the 24hR, and the percentage of agreement between the two methods was quite high for the majority of the food groups. The FV-GDR calculated from the DQQ correlated with the actual intake, although less strongly than the FV-GDR from 24hR. The DQQ is a promising low-burden, low-cost and simple tool to calculate FV-GDR and to monitor fruit and vegetable consumption at the population level. This provides the possibility of evaluating an important aspect of diet quality in low-resource settings.

Suboptimal diets characterised by low fruit and vegetable consumption are highly prevalent, especially in South Asia and Africa^(1,2)^. Assessing diet quality at the population level can help monitor and evaluate potential public health risk factors^(3)^. Simple-to-administer and low-priced indicators are essential tools to facilitate this and to overcome budget and resource limitations faced in many local and research settings^(4)^. At present, the majority of commonly used diet quality indicators are based on resource-intensive methodologies using quantitative data, such as weighted food records and quantitative 24-h recall (24hR)^(5)^. These dietary assessment methods require highly skilled interviewers and burdensome and time-consuming data collection processes^(6)^. Therefore, the use of these indicators is not always feasible in poor-resource settings, and intuitive, simple and low-burden indicators that reflect adequate intake are needed.

To address this gap, tools to collect population-level consumption data of food groups have been developed, such as a list-based or recall tool on the consumption of ten food groups to calculate the Food Group Dietary Diversity Score (FGDS). The FGDS is the sum of the number of food groups consumed, and a score ≥ 5 referred to as the minimum dietary diversity score^(7)^ is related to adequate micronutrient intake in women of reproductive age^(8,9)^. The FGDS includes four fruit and vegetable food groups: (1) dark green leafy vegetables, (2) other vitamin A-rich fruits and vegetables and (3) other vegetables, (4) other fruits. It is commonly used in settings where quantitative data collection is not possible^(10)^. However, the FGDS does not provide information on the risk of diet-related non-communicable diseases and has not been validated for men^(11)^. The recently developed Diet Quality Questionnaire (DQQ)^(12)^ is a simple and intuitive questionnaire, which enables gathering information on the consumption of twenty-nine food groups. In addition to calculating the FGDS, the DQQ can also be used to estimate the Global Dietary Recommendation (GDR) score^(4)^. The GDR score reflects adherence to global recommendations and the risk of non-communicable diseases and is based on the consumption of seventeen food groups, including six fruit and vegetable food groups (the fruit and vegetable component): (1) vitamin A-rich vegetables, (2) dark green leafy vegetables, (3) other vegetables, (4) vitamin A-rich fruits, (5) citrus and (6) other fruits.

With the recent growing interest in improving fruit and vegetable intake for better health and sustainability^(13,14)^, we hypothesised that the fruit and vegetable component of the GDR score (FV-GDR) can also be used to monitor and evaluate fruit and vegetable intake at the population level. This would provide a simple tool, requiring low training time and capability of the enumerators and simple processing of the data. To the best of our knowledge, only two studies have examined the validity of such a simple score to evaluate fruit and vegetable consumption. Herforth *et al.* showed good agreement between the FV-GDR and meeting the international fruit and vegetable recommendations of 400 g/d when derived from the same datasets^(4)^, but Hanley-Cook *et al.* showed low agreement in the proportion of women consuming fruits and vegetables of the FDGS compared with a weighed food record^(15)^.

In this paper, we investigate the relative validity of the FV-GDR collected with the DQQ to measure the actual intake of fruits and vegetables at the population level. We compared the FV-GDR of the DQQ to the results of a quantitative 24hR (as reference method) collected among a Vietnamese and a Nigerian study population to answer the following questions: (1) Do the two methods similarly estimate the proportion of food groups consumed at the population level? (2) Are fruit and vegetable intakes similarly correlated with FV-GDR irrespective whether the score is calculated from DQQ or from the reference method (24hR)?

## Method

### Study area and population

This study uses data collected as part of the ‘Fruit and Vegetable intake in Vietnam and Nigeria’ research project (FVN), which aimed to increase fruit and vegetable consumption of a low-income Vietnamese and Nigerian urban population. The FVN project focusing on women and men of reproductive age (19–49) at the start covered a total of 2 years. For the current study, we used FVN endline data, hence including 620 Vietnamese and 630 Nigerian women and men of 21–51 years of age. For each respondent, two dietary assessments were collected with at least 2 day in between each interview. In Vietnam (1240 observations) data were collected from October to December 2021 in Hanoi from two urban areas (Dong Da and Nam Tu Liem) and two peri-urban areas (Ha Dong and Thanh Tri). In Nigeria (1247 observations) data were collected in Ibadan from November to December 2021 from two urban areas (Abaeja and Apete) and two peri-urban areas (Bagadaje and Ariyibi).

### Ethical approval

This study was conducted according to the guidelines laid down in the Declaration of Helsinki, and all procedures involving human subjects were approved by the Hanoi Medical University Institutional Review Board in Hanoi (45-18/HMU-IRB) and the University of Ibadan/University College Hospital Ethical Review Committee (UI/UCH-ERC) in Nigeria (HNHREC/05/01/2008a). Written informed consent was obtained from all subjects.

### Dietary assessments

Dietary intake was assessed using a Dietary Quality Questionnaire (DQQ)^(16)^ and the multi-pass quantitative 24hR method^(17,18)^. The DQQ was collected digitally with KoboToolbox software^(19)^, while 24hR were collected on paper. Both dietary assessments were carried out during the same interview, always administering the DQQ first, followed by the 24hR, and in duplicate on a non-consecutive day. Trained local interviewers conducted interviews during home visits.

### Dietary Quality Questionnaire

The DQQ includes twenty-nine dichotomous questions (yes/no) about the food items and drinks consumed on the previous day or night, from when the respondent woke up the previous day to when she/he woke up on the day of the interview^(12)^. Each question includes a list of sentinel country-specific food items that reflects commonly consumed foods. These were identified from previous dietary assessments and key informant interviews with the local experts. Therefore, the specific sentinel food items included in the questionnaire differed between the two countries. For the present study, we considered only six questions regarding fruit and vegetables: (1) pro-vitamin A-rich orange vegetables; (2) dark green leafy vegetables; (3) other vegetables; (4) pro-vitamin A-rich fruits; (5) citrus and (6) other fruits (online Supplementary Tables S1–S2). Pro-vitamin A-rich orange vegetables and fruits are further referred to as ‘vitamin A-rich vegetables’ and ‘vitamin A-rich fruits’ for consistency with other publications on DQQ and GDR^(4,16,20)^.

### Quantitative 24-h Recall

We used the multi-pass quantitative 24hR method^(17,18)^ as a reference method for the validation of the DQQ. During home visits, respondents were asked, in the presence of the person who prepared the food, to recall all the foods and beverages they had consumed in and outside their home from waking up the day before the interview until waking up the day of the interview. Respondents were asked to describe in detail the composition of the mixed dishes, types of ingredients and cooking methods. To estimate the amount consumed, foods or ingredients still present in the household were weighed directly using an electronic kitchen scale (LP-B series for Vietnam; Camry EK5055 for Nigeria) with a precision of one decimal. If foods were unavailable, equivalent volumes of water or dry foods, such as rice or flour, or monetary values were used to estimate the amount consumed of each ingredient^(18)^. Then, the total volume of the cooked dish, the portion served and the portion left over were estimated using water. To calculate the actual intake in grams, waste factors, conversion factors and standardised recipes were applied.

### Variables construction

Fruit and vegetable food group consumption was defined as the consumption of each of the six fruit and vegetable food groups based on the GDR score. For each individual and recall day, a binary score for each food group consumed was extracted from the DQQ and constructed from the 24hR, being 1 if consumed and 0 if not consumed. For 24hR, all the consumed fruit and vegetable food items were aggregated into the same six food groups. Only foods consumed in quantities of at least 15 g were considered for comparison with the DQQ, as the DQQ focuses on sentinel (commonly consumed) fruits and vegetables, assuming the exclusion of food items consumed in small amounts^(21)^. The total FV-GDR score was defined as the sum of each fruit and vegetable food group consumed, ranging from 0 to 6, and was calculated based on the DQQ and 24hR. The total fruit and vegetable (grams per day) consumed was calculated from 24hR, summing the quantities of each fruit and vegetable item consumed.

### Data analysis

All analyses were conducted separately for Vietnam and Nigeria, and each dietary assessment for each respondent was considered a separate observation (descriptive analyses of single recall days are reported in online Supplementary Tables S3–S4). The difference in the population prevalence of each fruit and vegetable food group between DQQ and 24hR was calculated by subtracting the 24hR value from the DQQ value and was tested with linear mixed model to account for dependent observations. The difference in the mean total FV-GDR score between the DQQ and 24hR was calculated by subtracting the value of the 24hR value from the DQQ value and was tested with Wilcoxon rank test. To measure agreement between DQQ and 24hR, two-by-two tables were constructed. The proportion of misreporting in each fruit and vegetable group was calculated by investigating type I, false positive, and type II, false negative, errors. The false positive and false negative values were used to calculate the sensitivity and specificity of the consumption of each food group. Furthermore, we used linear mixed models to investigate the correlation of individual food groups among methods, total FV-GDR score, with actual fruit and vegetable intake from both DQQ and 24hR. We hypothesised that higher FV-GDR scores corresponded to higher fruit and vegetable intake using both methods. We tested for differences in the correlation coefficients between the two methods using Zou’s method^(22)^ and Hittner’s method^(23)^ as implemented in the R package cocor^(24)^ for dependent correlations with overlapping variables. Data analysis was performed in R^(25)^, and a significance level of *P* < 0·05 and a difference in proportion > 10 % were considered meaningful^(4,26)^.

## Results

### General population characteristics

More than half of the study population were women (65 % in Vietnam and 66 % in Nigeria) (online Supplementary Table S5). The mean values and standard deviations age of the total population were 38 (sd 7) years in Vietnam and 37 (sd 8) years in Nigeria, and the majority of respondents completed secondary education and above (41 % in Vietnam and 31 % in Nigeria). In Vietnam, 27 % of respondents were formally employed, more than a quarter was informally employed and 8 % owned a business. In Nigeria, a large proportion of the respondents were formally (42 %) or informally employed (38 %).

### Differences between Diet Quality Questionnaire and 24-h Recall

**Fig. 1.**
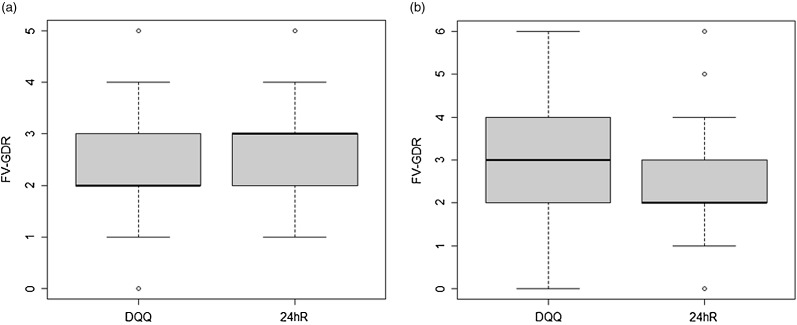
Box-and-whisker plots showing FV-GDR of the (a) Vietnamese and (b) Nigerian population as calculated from DQQ and 24hR, respectively. The bottom and top edge of the box represent the first and third quartiles (inter-quartile range); the bold line within the box represents the median and the ends of the bottom and top whiskers represent the 10th and 90th percentiles, respectively. FV-GDR, fruit and vegetable component of the Global Dietary Recommendation score; DQQ, Diet Quality Questionnaire; 24hR, 24-h recall.

In Vietnam, the median (inter-quartile range) of FV-GDR based on DQQ (2 (1), range 0–5) was lower than that based on the 24hR (3 (1), range 1–5) (*P* < 0·001) (Fig. 1(a)), mainly due to underreporting of the consumption of ‘other vegetables’ (20 percentage points) and ‘other fruits’ (18 percentage points) (Table 1). These were the only two food groups with a difference in proportion of > 10 %. In Nigeria, the median (inter-quartile range) of FV-GDR based on DQQ (3 (2), range 0–6) was higher than that of the 24hR (2 (1), range 0–6) (*P* < 0·001) (Fig. 1(b)), mainly due to the overreporting of ‘vitamin A-rich vegetables’ (41 percentage points) (Table 1). Histogram of the FV-GDR distribution (online Supplementary Fig. S1) and results from the sensitivity analysis for differences in proportion between methods including only the sentinel foods of the DQQ (online Supplementary Table S6) and all quantities consumed (online Supplementary Table S7) can be found in the Supplementary material.

**Table 1.** Proportions of Vietnamese and Nigerian populations having consumed each fruit and vegetable food group from DQQ and 24hR, and median intake based on 24hR (Numbers and percentages)

	Vietnam						Nigeria					
	DQQ (*n* 1240)		24hR (*n* 1240)				DQQ (*n* 1247)		24hR (*n* 1247)			
	*n*	%	*n*	%	Median intake, g	IQR	*n*	%	*n*	%	Median intake, g	IQR
Vitamin A-rich vegetables	167	13·5***	121	9·8	0	0	638	51·2***	125	10	0	0
Dark green leafy vegetables	1013	81·7	991	79·9	109·6	145	822	65·9***	702	56·3	21	48
Other vegetables	748	60·3***	989	79·8	124·6	207	1137	91·2***	1187	95·2	89·2	102
Vitamin A-rich fruits	123	9·9	129	10·4	0	0	116	9·3	121	9·7	0	0
Citrus	388	31·3	373	30·1	0	78	396	31·8*	385	30·9	0	140
Other fruits	452	36·5***	681	54·9	52·8	147	338	27·1	345	27·7	0	94
Total	1236	99·7	122	98·9	409·5	262	1221	97·9	1224	98·2	263·7	402

DQQ, Dietary Quality Questionnaire; 24hR, 24-h recalls; *n,* number of observations; median intake is reported in grams per day; IQR, interquartile range.

* *P* < 0·05; ***P < 0·001 for difference between DQQ and 24hR.

### Agreement in food group consumption between Diet Quality Questionnaire and 24-h Recall

In Vietnam, the percentage agreement coefficient of all food groups indicated high agreement between the DQQ and 24hR, ranging from 65 % to 90 % (Table 2). The DQQ correctly classified (e.g., true positive and true negative) ≥ 80 % of respondents who consumed ‘vitamin A-rich vegetables’, ‘dark green leafy vegetables’, ‘vitamin A-rich fruits’ and ‘citrus fruits’, and > 65 % of respondents who consumed ‘other vegetables’ and ‘other fruits’. Furthermore, DQQ had the highest sensitivity (e.g. true positive rate) for ‘dark green leafy vegetables’ (88 %) and the highest specificity (e.g., true negative rate) for ‘vitamin A-rich vegetables’ and all fruit groups (82–94 %). In Nigeria, the percentage agreement coefficient of all fruit food groups indicated very good agreement (87–98 %), whereas a poorer agreement (56 %) was found for ‘vitamin A-rich vegetables’ (Table 2). The DQQ correctly classified > 85 % of respondents as consuming all food groups, but only 56 % of respondents consumed ‘vitamin A-rich vegetables’. High sensitivity was found for all groups (88–99 %), and the specificity was higher for fruit groups (0·98–0·99) compared with the vegetable groups (0·53–0·74).

**Table 2.** Misreporting and agreement of food groups from DQQ and 24hR in the Vietnamese and Nigerian study population

	Misreporting				Agreement statistics
	% FP	% FN	Sensitivity	Specificity	% agreement
Vietnam					
Vitamin A-rich vegetables	8·5	4·8	0·5	0·90	90·2
Dark green leafy vegetables	11	9·3	0·88	0·45	79·7
Other vegetables	6·5	26	0·67	0·68	67·5
Vitamin A-rich fruits	5·2	5·7	0·45	0·94	89·0
Citrus	11	9·8	0·68	0·84	79·3
Other fruits	8·1	26·6	0·52	0·82	65·2
Nigeria					
Vitamin A-rich vegetables	42·3	1·2	0·88	0·53	56·4
Dark green leafy vegetables	11·2	1·6	0·97	0·74	87·2
Other vegetables	2	6	0·94	0·58	93·0
Vitamin A-rich fruits	0·5	0·9	0·91	0·99	98·6
Citrus	1·4	0·6	0·98	0·98	98·0
Other fruits	1·3	1·8	0·99	0·99	96·9

DQQ, Diet Quality Questionnaire; 24hR, 24-h recall; FP, false positive, type I error; FN, false negative, type II error. Coefficient agreement formula: ((a + d)/n) × 100, where *a* is the number of observations not having consumed the food group in both DQQ and 24hR, *d* is the number of observations having consumed the food group in both DQQ and 24hR, and *n* is the total number of observations.

### Misreporting of food groups by Diet Quality Questionnaire compared with 24-h recall

In Vietnam, type I measurement errors (false positive) occurred mainly from the overreporting of ‘dark green leafy vegetables’ (11 %) and ‘citrus’ (11 %). Measurement error type II (false negative) occurred from underreporting 27 % of ‘other vegetables’ and 27 % of ‘other fruits’ (Table 2). In Nigeria, type I errors mainly occurred from overreporting 42 % of ‘vitamin A-rich vegetables’ and 11 % of ‘dark green leafy vegetables’, whereas underreporting was low for all food groups (0·6–1·8 %) (Table 2).

### Correlations of food groups and total score

**Fig. 2.**
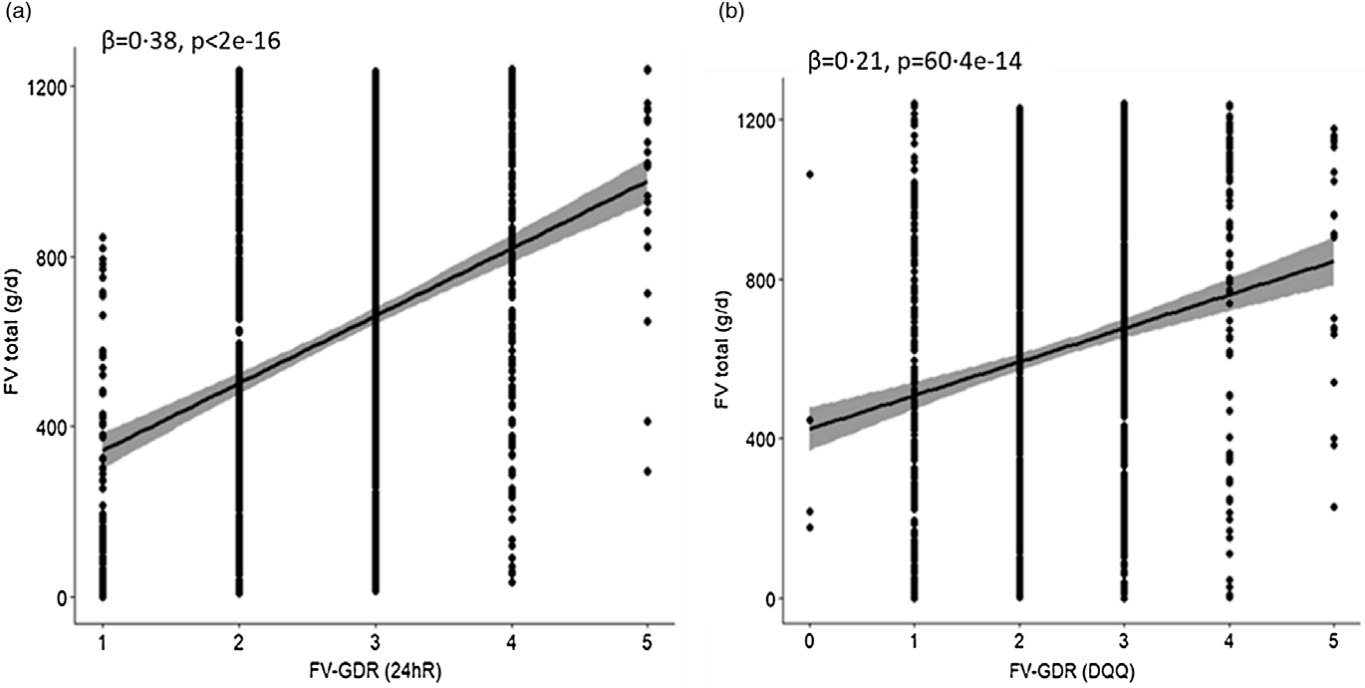
Regression of fruit and vegetable intake on FV-GDR score calculated from (a) 24hR and (b) DQQ for Vietnam. x-axis = FV-GDR; y-axis = fruit and vegetable (FV) intake in grams per day. FV-GDR, fruit and vegetable component of the Global Dietary Recommendation score; DQQ, Diet Quality Questionnaire; 24hR, 24-h recall.

**Fig. 3.**
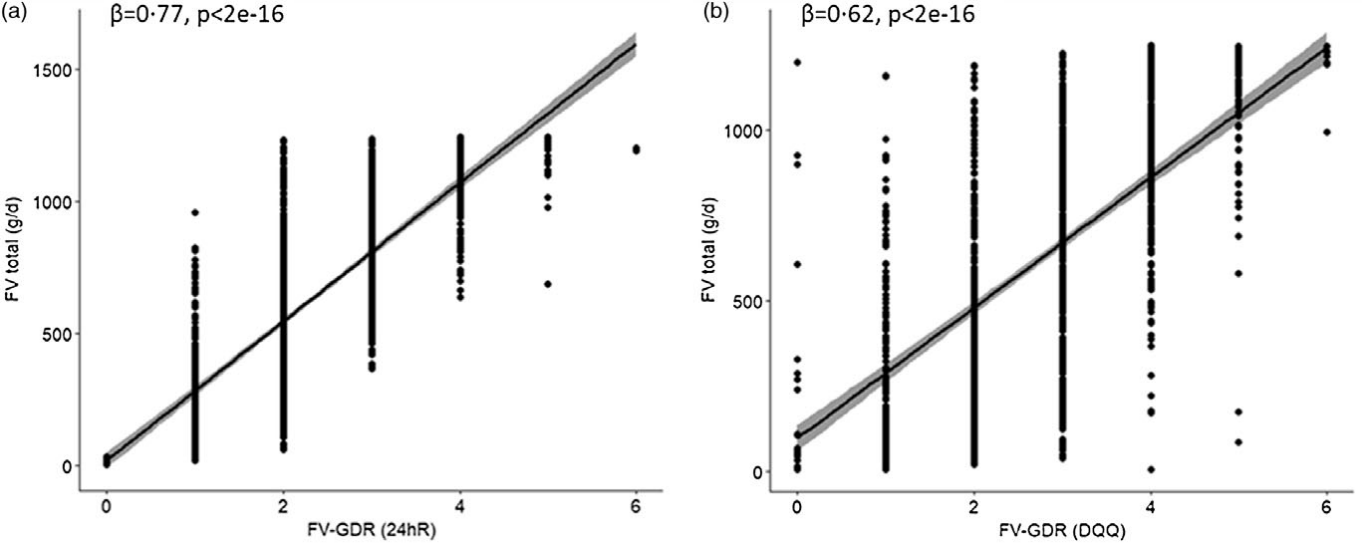
Regression of fruit and vegetable intake and FV-GDR score calculated from (a) 24hR and (b) DQQ for Nigeria. x-axis = FV-GDR; y-axis = fruit and vegetable (FV) intake in grams per day. FV-GDR, fruit and vegetable component of the Global Dietary Recommendation score; DQQ, Diet Quality Questionnaire; 24hR, 24-h recall.

In Vietnam, the correlations of food group consumption between the DQQ and 24hR ranged from 0·28 to 0·51 with *P* < 0·001 for all groups (Table 3). A *β*_st_ = 0·38 (95 % CI 0·33, 0·43) was found between the total FV-GDR calculated from the 24hR and the total fruit and vegetable intake (Fig. 2(a)). The correlation was lower between FV-GDR from the DQQ and total fruit and vegetable intake (*β*_st_ = 0·21, 95 % CI 0·16, 0·27) (Fig. 2(b)). Furthermore, the two correlations (DQQ *v*. 24hR) were found to be different (z = 5·59, 95 % CI 0·11, 0·23). In Nigeria, a strong correlation between DQQ and 24hR was found for all fruit groups (0·92–0·95, *P* < 0·001) and ‘dark green leafy vegetables’ (0·71, *P* < 0·001) (Table 3). In contrast, *β*_st_ = 0·24 (95 % CI 0·19, 0·30) was found for ‘vitamin A-rich vegetables’ and *β*_st_ = 0·39 (95 % CI 0·34, 0·44) for ‘other vegetables’. A high correlation was found between FV-GDR and total fruit and vegetable intake, but it was lower for the FV-GDR calculated from the DQQ (*β*_st_ = 0·62, 95 % CI 0·58, 0·66) than for the 24hR (*β*_st_ = 0·77, 95 % CI 0·73, 0·80) (Fig. 3(a) and (b)). Also for Nigeria, the two correlations (DQQ *v*. 24hR) were found to be different (z = 7·86, 95 % CI 0·11, 0·19).

**Table 3.** Associations between DQQ and 24hR per each food group for the Vietnamese and the Nigerian study population (Beta-coefficients and 95 % confidence intervals)

	Vietnam		Nigeria	
	*β*_st_	95 % CI	*β*_st_	95 % CI
Vitamin A-rich vegetables	0·33	0·28, 0·38	0·24	0·19, 0·30
Dark green leafy vegetables	0·34	0·29, 0·40	0·71	0·71, 0·78
Other vegetables	0·28	0·22, 0·33	0·39	0·34, 0·44
Vitamin A-rich fruits	0·40	0·35, 0·45	0·92	0·90, 0·94
Citrus	0·51	0·46, 0·56	0·95	0·94, 0·97
Other fruits	0·33	0·28, 0·38	0·92	0·90, 0·94

DQQ, Diet Quality Questionnaire; 24hR, 24-h recall; *β*_st_, linear mixed model standardised estimates; for all correlations *P* < 0·001.

## Discussion

In general, this study suggests that the FV-GDR calculated from the Dietary Quality Questionnaire (DQQ) is a good indicator of fruit and vegetable intake at the population level. In two study countries, Vietnam and Nigeria, we found that the DQQ performs well in the estimation of consumption of total fruits and vegetables, with small differences in total FV-GDR compared with the reference method (24hR). The percentage of agreement in FV-GDR between the two methods was shown to be quite high for the majority of the fruit and vegetable food groups, and the FV-GDR from the DQQ correlated with the actual amount consumed, although less strongly than the FV-GDR calculated from the 24hR.

However, in both countries, the consumption of some specific food groups was underestimated (‘other vegetables’ and ‘other fruits’ in Vietnam) or overestimated (‘vitamin A-rich vegetables’ in Nigeria). Compared with the reference method, the DQQ seems to underestimate the intake of food groups that reflect a high variety of food items. For instance, the list of sentinel foods in the DQQ for ‘other vegetables’ did not capture some foods commonly consumed by the study population (bamboo shoots and mushrooms) or those consumed in specific seasons of the year (eggplants and kohlrabi). Moreover, the vegetables classified in the ‘other vegetables’ group consumed in mixed dishes or prepared out of home could have been underestimated with the DQQ because of the difficulty of recognising, remembering and measuring all ingredients^(27)^, while being included as standard recipes in the processing of 24hR data. Moreover, as expected, the proportions of food group consumption became more similar when the proportion calculated from the 24hR was limited to the sentinel food items as included in the DQQ. Increasing the number of sentinel foods and the number of questions for food groups with a large variety may increase the probability of capturing the majority of food items consumed and reduce this underestimation. Furthermore, the lists of sentinel foods must be closely assessed to ensure that they fully capture the majority of food items available and actually consumed. Since the DQQ has been designed to assess the quality of diet at the country level, specific food habits of sub-populations may be missed. In our study, this issue may explain the underestimation of some food groups in Vietnam because the DQQ for the whole of Vietnam was used, but only a sub-population from the city of Hanoi was assessed. In contrast, the Nigerian DQQ used in this study was tailored to the target population (FVN project). Therefore, the DQQ available from the Dietary Quality Project present slightly different lists of sentinel foods. Overall, adapting the questionnaire based on the context, sub-population targeted, specific region or period of the year of interest will increase the accuracy of estimation of intake using the DQQ.

In contrast, the DQQ seemed to overestimate food groups for which items were consumed in small portions. This could explain the large difference in the proportion of ‘vitamin A-rich vegetables’ consumed in Nigeria. This food group includes *tatashe*, one of the ingredients used to prepare a commonly consumed tomato-based sauce, which is consumed in small quantities. With the DQQ, respondents answered whether the listed foods were consumed, regardless of the quantity actually consumed. When we assessed the overestimation of ‘vitamin A-rich vegetables’ in the DQQ compared with 24hR without excluding foods consumed <15 g, the overestimation was indeed reduced. Additionally, the underreporting of the 24hR for episodically consumed foods, such as fruits, shown in studies in low-income countries, could also possibly explain the overestimation of the DQQ found in this study^(5,28–30)^. In other words, respondents may have correctly reported their intake when probed by the DQQ list of sentinel foods, compared with the 24hR where they were prone to forget episodically consumed foods in an open recall.

Moreover, we found that the DQQ was more accurate in estimating fruit intake than vegetable intake. This is probably due to the modality of usual fruit consumption. We observed that fruits are commonly consumed per item and are rarely consumed in mixed dishes. Therefore, they are rarely consumed in amounts of < 15 g, reducing the possibility of recall bias related to recalling several ingredients included in a dish. Second, because fruits are not commonly consumed, they are rarely consumed more than once a day or more than one type of fruit within the same food group^(31)^; thus, they are more easily remembered. This reduces the risk of underestimation and overestimation, although in contrast with Hanley-Cook’s *et al.* findings^(15)^. Third, especially in Nigeria, the varieties available and actually consumed are relatively low and, therefore, well represented in the DQQ sentinel food list. This may explain the high agreement in the consumption of all fruit food groups in this study in Nigeria. However, the high variety of fruits available and commonly consumed in Vietnam will increase the chance that some fruits are not captured in the DQQ, which could explain the lowest percentage of agreement for other fruit food groups found in our study in Vietnam. Therefore, including in the DQQ more questions for the food groups with a large variety of food items eaten, considering seasonal availability of items, and excluding from the list of sentinel foods, the items that are mainly consumed in low amounts (e.g. mixed in recipes, sauces) may contribute to addressing the above-mentioned causes of under- and overestimation of some fruit and vegetable food groups.

Regarding the total FV-GDR, the DQQ seems to be a promising tool that can be used to evaluate and monitor fruit and vegetable intake at the population level. Although we found a statistically significant difference in the score between the two methods in our two study countries, we consider this difference not relevant at the population level and from a public health perspective. Considering that we propose the FV-GDR to assess and monitor fruit and vegetable intake in large populations, the evaluation of total fruit and vegetable intake would be similar between the two methods.

As hypothesised, FV-GDR positively correlated with the actual intake of fruits and vegetables in both methods. This correlation was stronger in Nigeria than in Vietnam. As expected, the correlation was lower when the FV-GDR was calculated from the DQQ, as it includes a limited list of food items compared with an open recall^(32)^. However, the positive correlation indicates that a higher FV-GDR score based on the DQQ indicates a higher intake of fruits and vegetables but does not indicate whether that intake is sufficient, that is, meets the WHO recommendations of 400 g or more. At the individual level, Herforth *et al.* proposed a cut-off point for consuming at least three fruit and vegetable food groups, indicating adherence to the WHO recommendations^(4)^. However, the proposed cut-off point has not been validated for any Asian or African country but was based on data from the USA and Brazil^(4)^. Based on our data, there is a large difference (> 20 percentage points) in the proportion of respondents above this cut-off point between the DQQ and 24hR, both in Vietnam and Nigeria, and we found a cut-off of 0·5 (Vietnam) and 1·5 (Nigeria). Although the identification of a global cut-off point for fruit and vegetable food group intake would provide another useful indicator of adherence to diet quality recommendations, our data possibly suggest that optimal cut-off might vary across countries. Therefore, more research is needed to formulate such a global cut-off point.

The large number of observations and the administration of the two tools separately in the same interview allowed us to better estimate the validity of the fruit and vegetable component of the DQQ to assess fruit and vegetable actual intake at the population level^(33)^. However, correlated measurement error might have inflated the correlation between the methods because both rely on the memory of the respondents. On the other hand, the correlated measurement error when investigating the correlation between intake (24hR) and score (DQQ) is lower than that obtained using the 24hR dataset for calculating the FV-GDR^(33)^. Administration of the DQQ always before the 24hR prevented influencing the DQQ answers of the respondent through a 24hR, assuming that the DQQ would not affect the 24hR because it is a simpler method. In this study, we used 24hR as the reference method, although it is not the gold standard. Weighed food records could have provided a better estimation of the actual intake, but considering the budget and resources available for the study, 24hR was chosen as the best method^(34)^. In addition, our results can be generalised only to low-income, mainly female populations in the context of urban and peri-urban Vietnam and Nigeria, and further investigation in other contexts (e.g. other countries, rural areas and minorities with different dietary habits) is needed to generalise the validity of the FV-GDR score for monitoring fruit and vegetable intake at the population level.

To conclude, the DQQ is a very promising tool for calculating the FV-GDR and monitoring total fruit and vegetable consumption at the population level. It provides the possibility of using a low-burden, low-cost and simple-to-use tool to assess fruit and vegetable intake in low-resource settings.

## Supplementary material

For supplementary material accompanying this paper visit https://doi.org/10.1017/S0007114523001125. click here to view supplementary material
