# Supplementary material for: A simple fruit and vegetable score is a valid tool to assess actual fruit and vegetable intake
Source: Br J Nutr. 2023 May 5;130(11):1942–9. doi: 10.1017/S0007114523001125 (PMC10632721; doi:10.1017/S0007114523001125)
Supplement: Supplementary file 1 [file S0007114523001125sup001.docx]

**Supplementary material**

Table S1. Fruit and vegetable questions of the Dietary Quality Questionnaire administrated in Vietnam

| **Do not** read the food group names | **Yesterday did you eat any of the following vegetables?** | (circle answer) |
| --- | --- | --- |
| *Vitamin A rich orange vegetables* | Carrot *(ca rot),* pumpkin *(bi ngo),* orange-fleshed sweet potato or purple-flesh sweet potato? | YES or NO |
| *Dark green leafy vegetables* | Morning glory/water spinach *(rau muong),* katuk *(rau ngót),* sweet potato leaves *(rau khoai lang)*, ceylon spinach (*rau mồng tơi)*, pumpkin leaves (rau bí), chayote leaves (ngọn su su), or jute potherb (rau đay)? | YES or NO |
| *Dark green leafy vegetables* | Broccoli (Súp lơ xanh), amaranth *(rau den)*, watercress *(cai soong)*, napa cabbage *(cai thao)*, mustard greens *(cai xanh)*, cassava leaves, or crown daisy (cải cúc)? | YES or NO |
| *Other vegetables* | Cabbage *(cai bap)*, lettuce (rau xà lách), mung bean sprout, common bean (dau cove), cauliflower (sup lo trang), Pepper (ot chuong), or tomato (ca chua)? | YES or NO |
| *Other vegetables* | Calabash (bau), ashgourd/waxgourd (bi xanh), gourd (muop), bitter melon (muop dang), Cucumber (dua leo), White radish (cu cai trang), or chayote (su su)? | YES or NO |
|  | **Yesterday did you eat any of the following fruits?** | (circle answer) |
| *Vitamin A rich fruits* | Ripe mango *(xoai chin)*, ripe papaya, passion fruit *(chanh leo)*, or persimmon *(hong mem)*? | YES or NO |
| *Citrus* | Orange *(cam)*, pomelo, grapefruit, tangerine, clementine, or mandarin? | YES or NO |
| *Other fruits* | Banana, pineapple (qua dua), avocado (qua bo), watermelon (dua hau), guava *(oi)*, grape (*nho ngot)*, or mangosteen (mang cut)? | YES or NO |
| *Other fruits* | Rambutan (chom chom), longan (nhan), litchi (qua vai), dragonfruit (thanh long), jackfruit (mit), durian (sau rieng), or sugar apple (na)? | YES or NO |

Table S2. Fruit and vegetable questions of the Dietary Quality Questionnaire administrated in Nigeria

| **Do not** read the food group names | **Yesterday did you eat any of the following vegetables?** | (circle answer) |
| --- | --- | --- |
| *Vitamin A rich orange vegetables* | Carrot, pumpkin, tatashe, or orange or yellow-fleshed sweet potato? | YES or NO |
| *Dark green leafy vegetables* | Ewedu, amaranthus leaves, water leaf, shoko, bitter leaf, ugu, osun or spinach? | YES or NO |
| *Other vegetables* | Tomato, cucumber, okro or garden egg? | YES or NO |
|  | **Yesterday did you eat any of the following fruits?** | (circle answer) |
| *Vitamin A rich fruits* | Ripe mango, ripe pawpaw, locust bean fruit, hog plum, or bush mango fruit? | YES or NO |
| *Citrus* | Orange, tangerine, tangelo or grapefruit? | YES or NO |
| *Other fruits* | Banana, agbalumo, watermelon, apple, avocado, coconut, pineapple, guava or cashew fruit? | YES or NO |

Table S3. Proportions of food group consumed from DQQ and 24hR, and intakes from 24hR for recall 1 and recall 2, Vietnam

|  | **Vietnam** | | | | | | | | | |
| --- | --- | --- | --- | --- | --- | --- | --- | --- | --- | --- |
|  | **Recall 1** | | | | | **Recall 2** | | | | |
|  | **DQQ**  **(n=620)** | | **24hR**  **(n=620)** | | | **DQQ**  **(n=620)** | | **24hR**  **(n=620)** | | |
|  | **n** | **%** | **n** | **%** | **Median intake, g (IQR)** | **n** | **%** | **n** | **%** | **Median intake, g (IQR)** |
| Vitamin A-rich vegetables | 86 | 13.9 | 71 | 11.5 | 0 (0) | 81 | 13.1 | 81 | 13.1 | 0 (0) |
| Dark green leafy vegetables | 512 | 82.6 | 500 | 80.6 | 118.5 (145) | 501 | 80.8 | 497 | 80.2 | 100 (145) |
| Other vegetables | 375 | 60.5 | 528 | 85.2 | 117.2 (208.3) | 373 | 60.2 | 527 | 85 | 130.7 (205.3) |
| Vitamin A-rich fruits | 55 | 8.9 | 61 | 9.8 | 0 (0) | 68 | 11 | 68 | 11 | 0 (0) |
| Citrus | 225 | 36.3 | 221 | 35.6 | 0 (97.3) | 163 | 26.3 | 160 | 25.8 | 0 (40.7) |
| Other fruits | 215 | 34.7 | 347 | 56 | 54.7 (148.5) | 237 | 38.2 | 349 | 56.3 | 49.3 (146.8) |
| FV-GDR, mean (sd),  Min-max | 2.4  0 | (0.9)  5 | 2.7  1 | (0.8)  5 |  | 2.3  0 | (0.9)  5 | 2.6  1 | (0.8)  5 |  |
| DQQ **=** Dietary Quality Questionnaire; 24hR = 24hour recalls; n = number of observations; Median intake is reported in grams per day; IQR = interquartile range; ******p* < 0.05; ** *p* < 0.01; *** *p <* 0.001 for difference between DQQ and 24hR. | | | | | | | | | | |

Table S4. Proportions of food group consumed from DQQ and 24hR, and intakes from 24hR for recall 1 and recall 2, Nigeria

|  | **Nigeria** | | | | | | | | | |
| --- | --- | --- | --- | --- | --- | --- | --- | --- | --- | --- |
|  | **Recall 1** | | | | | **Recall 2** | | | | |
|  | **DQQ**  **(n=629)** | | **24hR**  **(n=629)** | | | **DQQ**  **(n=618)** | | **24hR**  **(n=618)** | | |
|  | **n** | **%** | **n** | **%** | **Median intake, g (IQR)** | **n** | **%** | **n** | **%** | **Median intake, g (IQR)** |
| Vitamin A-rich vegetables | 317 | 50.4 | 68 | 10.8 | 0 (0) | 321 | 51.9 | 57 | 9.2 | 0 (0) |
| Dark green leafy vegetables | 438 | 69.6 | 370 | 58.7 | 23 (48) | 384 | 62.1 | 332 | 53.8 | 19 (48) |
| Other vegetables | 573 | 91.1 | 596 | 94.6 | 90.8 (114.6) | 564 | 91.3 | 591 | 95.8 | 88 (90.5) |
| Vitamin A-rich fruits | 51 | 8.1 | 55 | 8.7 | 0 (0) | 65 | 10.5 | 66 | 10.7 | 0 (0) |
| Citrus | 222 | 35.3 | 212 | 33.7 | 0 (170) | 174 | 28.2 | 173 | 28 | 0 (140) |
| Other fruits | 176 | 28 | 179 | 28.4 | 0 (94) | 162 | 26.2 | 166 | 26.9 | 0 (94) |
| FV-GDR, mean (sd),  Min-max | 2.8  0 | (1.2)  6 | 2.3  0 | (1.1)  6 |  | 2.7  0 | (1.2)  6 | 2.2  0 | (1.1)  6 |  |
| DQQ **=** Dietary Quality Questionnaire; 24hR = 24hour recalls; n = number of observations; Median intake is reported in grams per day; IQR = interquartile range; ******p* < 0.05; ** *p* < 0.01; *** *p <* 0.001 for difference between DQQ and 24hR. | | | | | | | | | | |

Table S5. General characteristics of the study population, Vietnam and Nigeria.

|  | **Vietnam** | **Nigeria** |
| --- | --- | --- |
| **n** | 620 | 630 |
| **Age, mean years (sd)** | 38.4 (7.4) | 37.3 (7.7) |
| **Women, % (n)** | 64.9 (381) | 65.9 (415) |
| **Educational level, % (n)**  no education  primary school  secondary school  above secondary school | 0.5 (3)  4.1 (24)  27.1 (105)  41 (241) | 4.3 (27)  21 (132)  12.9 (81)  31.1 (196) |
| **Occupation, % (n)**  Skilled employment  Unskilled employment  Own business  Unemployed | 26.6 (156)  26.6 (156)  7.5 (64)  4 (24) | 41.6 (262)  38.1 (240)  12.5 (79)  1.4 (9) |

a) b)


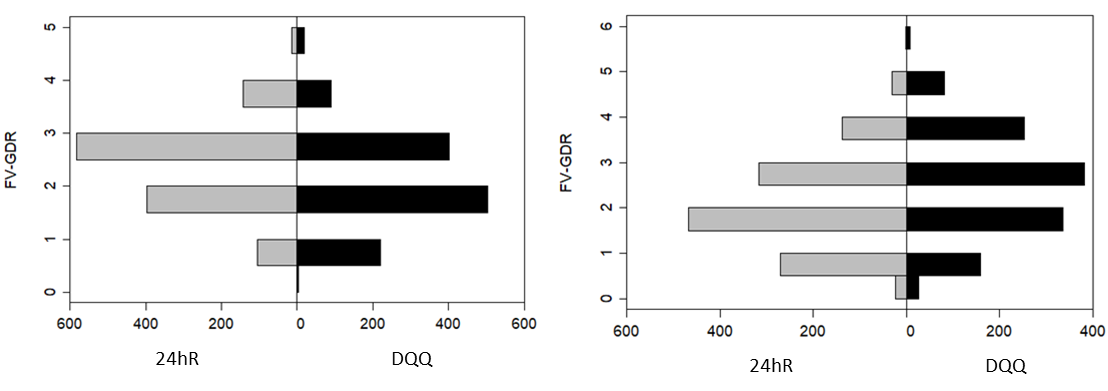


Figure S1. Back-to-back plots showing the distribution of FV-GDR in the a) Vietnamese and b) Nigerian study population calculated from 24hR (gray) and DQQ (black), respectively.

Table S6. Proportion of food groups consumed calculated from 24hR including only sentinel foods of the DQQ.

|  | Vietnam | | Nigeria | |
| --- | --- | --- | --- | --- |
|  | n | % | n | % |
| Vitamin A rich vegetables | 150 | 12.1 | 265 | 29.7 |
| Dark green leafy vegetables | 989 | 79.8 | 818 | 66 |
| Other vegetables | 869 | 70.1 | 1227 | 99 |
| Vitamin A riche fruits | 128 | 10.3 | 121 | 9.8 |
| Citrus | 397 | 32 | 386 | 31.2 |
| Other fruits | 425 | 34.3 | 345 | 27.8 |

Table S7. Proportion of food groups consumed when all quantities are included in the analysis for Vietnam and Nigeria

|  | **Vietnam** | | | | **Nigeria** | | | |
| --- | --- | --- | --- | --- | --- | --- | --- | --- |
|  | **DQQ**  **(n=1240)** | | **24hR**  **(n=1240)** | | **DQQ**  **(n=1248)** | | **24hR**  **(n=1248)** | |
|  | **n** | **%** | **n** | **%** | **n** | **%** | **n** | **%** |
| Vitamin A rich vegetables | 167 | 13.5 | 152 | 12.3 | 638 | 51.1 | 227 | 18.3 |
| Dark green leafy vegetables | 1013 | 81.7 | 997 | 80.4 | 823 | 65.9 | 818 | 66 |
| Other vegetables | 748 | 60.3 | 1055 | 85.1 | 1138 | 91.2 | 1227 | 99 |
| Vitamin A rich fruits | 123 | 9.9 | 129 | 10.4 | 116 | 9.3 | 121 | 9.8 |
| Citrus | 388 | 31.3 | 381 | 30.7 | 396 | 31.7 | 386 | 31.2 |
| Other fruits | 452 | 36.5 | 696 | 56.1 | 338 | 27.1 | 345 | 27.8 |
|  | **Mean** | **(sd)** |  |  | **Mean** | **(sd)** |  |  |
| FV-GDR | 2.33 | (0.92) |  |  | 2.8 | (1.20) |  |  |
| (min – max) | (0-5) |  |  |  | (0-6) |  |  |  |

DQQ **=** Dietary Quality Questionnaire; 24hR = 24hour recalls
